# Postpartum ovarian vein thrombophlebitis presenting as vaginal bleeding: A case report

**DOI:** 10.1097/MD.0000000000024632

**Published:** 2021-02-26

**Authors:** Tsai-Lien Chiang, Chuan-Yaw Chang, Jiann Ruey Ong

**Affiliations:** a Department of Emergency Medicine; b Department of Obstetrics and Gynecology, Shuang-Ho Hospital, Taipei Medical University, Taiwan.

**Keywords:** abdominal pain, ovarian vein thrombophlebitis, postpartum vaginal bleeding

## Abstract

**Rationale:**

Postpartum ovarian vein thrombophlebitis (POVT) is a rare condition, and it can lead to severe complications and mortality. Here we report a patient who presented with vaginal bleeding and the diagnosis of POVT was confirmed by imaging.

**Patient concerns:**

A 38-year-old postpartum woman without remarkable medical history presented with vaginal bleeding and lower abdominal pain.

**Diagnoses:**

The diagnosis was confirmed by computed tomography scan marked by a thrombus mass involving the right ovarian vein and inferior vena cava.

**Interventions:**

The patient was treated with intravenous antibiotics and low-molecular-weight heparin.

**Outcomes:**

The patient recovered smoothly without complications.

**Lessons:**

We should pay high attention to the recognition and management of POVT to prevent morbidity and mortality.

## 1 Introduction

Postpartum ovarian vein thrombophlebitis (POVT) is a rare but serious condition that requires medical attention.^[1]^ The primary recognition of POVT may be made from a combination of clinical features, namely pelvic pain, fever, and a right abdominal mass.^[2–4]^ Previous literatures reported that POVT may lead to fatal complications including systemic sepsis and pulmonary embolism.^[2,5]^ However the diagnosis of PVOT is often difficult because the common clinical presentations are mostly nonspecific, and it is hard to differentiate it from other common causes of postpartum abdominal pain such as pelvic endometritis, appendicitis, and acute pyelonephritis.

We describe a patient with POVT presenting to our emergency department (ED) with vaginal bleeding. The combination of bleeding with a hypercoagulable state was challenging to diagnose.

## 2 Case report

A 38-year-old woman (gravid 0, para 0) with an unremarkable medical history presented to the ED with vaginal bleeding and 1 day history of lower abdominal pain. The patient had given birth by cesarean section 13 days prior. Her antepartum course, labor, and delivery were uncomplicated.

On presentation, the patient was afebrile, and physical examination revealed right lower quadrant tenderness with no peritoneal signs or palpable mass.

Laboratory tests revealed anemia (hemoglobin level: of 8.0 g/dL), leukocytosis with neutrophilia (21,900 cells/uL), and an elevated C-reactive protein level (8.90 mg/dL).

**Figure 1:**
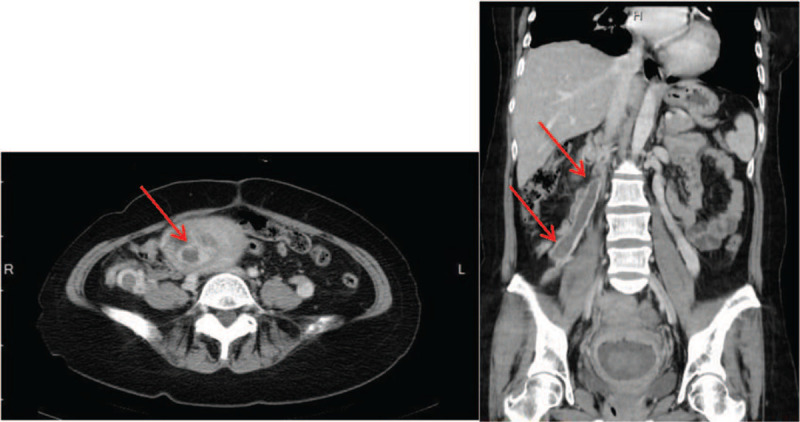
Contrast-enhanced computed tomography scan of the patient.

Vaginal ultrasound revealed a heterogeneous mass near the cervix, and an abscess was suspected. Computed tomography (CT) showed right ovarian vein thrombosis with a small extension into the inferior vena cava (IVC) and an eccentric intracavitary mass lesion at the dome of the uterus, suspected to be retained products of conception (Fig. 1).

The patient was treated with broad-spectrum antibiotics, oxytocin, low-molecular-weight heparin, and intravenous (IV) fluid supplement. Therapeutic dilatation and curettage was performed on the next day, during which blood clots with fragments of placenta-like tissue were evacuated and retrieved.

The clinical conditions improved during the hospitalization and all the interventions were tolerated well without apparent clinical complications. The surgical wound was clean and the patient was able to tolerate oral intake after dilatation and curettage procedure. There was no more fever, chest or abdominal discomfort, vaginal bleeding, voiding difficulty noted after therapy. The patient was discharged within 5 days of admission.

## 3 Discussion

OVT is a rare condition most often seen in postpartum women, and the proportion of symptomatic patients is as low as 0.01% to 0.05% postdelivery.^[2,6]^ Women undergoing cesarean delivery have a higher risk than those undergoing vaginal delivery.^[7]^ Most symptomatic POVT occur in the first 10 days after delivery but can also occur as late as 1 month postpartum.^[2]^

Unfortunately, the symptoms of POVT are mostly nonspecific. The classic triad of OVT symptoms consists of pelvic pain, fever, and a right abdominal mass; however, the symptoms of patients may only vaguely fit into this triad.^[8]^ Approximately 80% of patients with POVT present with fever, but only 30% to 50% experience abdominal pain.^[9]^ Pyelonephritis, tubo-ovarian abscess with or without torsion, endometritis, chorioamnionitis, infected abdomino-pelvic hematoma, diverticulitis, and appendicitis all may present similarly to POVT symptoms. Thus, both a high index of suspicion and further imaging are required to make a diagnosis.

The underlying pathophysiology of POVT has been related to Virchow's triad, which is composed of endothelial damage, venous stasis, and a hypercoagulable state.^[8]^ Pregnancy is a classic example of Virchow's triad. Hypercoagulability following delivery is widely thought to be a mechanism to protect women from bleeding problems during and after childbirth.

Common causes of postpartum hemorrhage include uterine atony, trauma, retained placenta, and coagulopathy. Uterine atony is responsible for most postpartum hemorrhage cases.^[10]^ Women who undergo cesarean section have higher rates of retained placenta (3.44%) than those who undergo vaginal delivery (1.96%).^[11]^

Our case illustrates an unusual initial presentation of POVT with vaginal bleeding upon arrival at the ED. Diagnostic confusion arises from the overlapping of the different mechanisms of bleeding and hypercoagulopathy. However, there is a paucity of literature describing cases such as ours, in which a hypercoagulable state of OVT presented simultaneously with a bleeding state in the absence of labor trauma, coagulation disorders, or postpartum blood product resuscitations.

Imaging modalities such as Doppler sonography, contrast CT scans, and magnetic resonance angiography (MRA) play an important role in the diagnosis of POVT.^[12]^ Among the imaging tools mentioned, ultrasound remains noninvasive and easy to use. Absence of blood flow and a hypoechoic tubular mass cephalad to the ovary and extending into the IVC may be identified on ultrasound. CT and MRA are more sensitive and specific than ultrasound for the diagnosis of POVT.^[13]^ Features identifiable on a CT scan include the thick-walled and enlarged ovarian vein with central tubular hypodensity and rim enhancement.^[14]^ MRA findings are similar to those of CT, but the sensitivity of MRA is considered to be nearly 100%.^[11]^ Regrettably, MRA is not as immediately available as CT in all ED settings.

Laboratory evaluation can usually reveal leukocytosis and an elevated C-reactive protein level, although anemia has also been reported in some cases.^[8]^ In most OVT cases, positive cultures are not found.^[8]^

Life-threatening complications of OVT, particularly pulmonary embolism and sepsis, may be precipitated by delays in diagnosis and treatment. If left untreated, the incidence of pulmonary embolism is reported to be 25%, with mortality reaching 4%.^[15]^

The mainstay of treatment in the current literature includes IV heparin and antibiotics. An initial 7-to-10-day course of IV heparin is followed by warfarin, and warfarin use may be prolonged to 3 months for thrombi extending into the IVC.^[4]^ Low-molecular-weight heparin is also worth considering because it is understood to be as effective as unfractionated heparin.^[16]^ Conversely, no evidence has been proposed to support the use of nonvitamin K antagonist oral anticoagulants for treating OVT.^[17]^

There are some limitations in our report. First, this is a case report, thus it is a retrospective design and is hard to establish cause-effect relationship. Furthermore, the initial presentation of our case was unusual, hence, there is difficulty to expand our experience to the general population.

High clinical suspicion of the diagnosis should be applied to avoid potentially life-threatening complications such as ovarian infarction, pulmonary embolism, ovarian abscess, and uterine necrosis.^[8,18]^ Early recognition and treatment may help to prevent morbidity.

## Author contributions

**Conceptualization:** Tsai-Lien Chiang, Jiann Ruey Ong.

**Supervision:** Chuan-Yaw Chang, Jiann Ruey Ong.

**Writing – original draft:** Tsai-Lien Chiang.

**Writing – review & editing:** Tsai-Lien Chiang, Chuan-Yaw Chang, Jiann Ruey Ong.
